# Evaluation of the usability of various rapid antibody tests in the diagnostic application for COVID-19

**DOI:** 10.1177/0004563220984827

**Published:** 2021-01-07

**Authors:** Yoshifumi Uwamino, Masatoshi Wakui, Wataru Aoki, Toshinobu Kurafuji, Emmy Yanagita, Maasa Morita, Mika Nagata, Rika Inose, Masayo Noguchi, Hiromitsu Yokota, Naoki Hasegawa, Hideyuki Saya, Mitsuru Murata, for the Keio Donner Project Team

**Affiliations:** 1 Department of Laboratory Medicine, Keio University School of Medicine, Tokyo, Japan; 2 Department of Infectious Diseases, Keio University School of Medicine, Tokyo, Japan; 3 Clinical Laboratory, Keio University Hospital, Tokyo, Japan; 4 Division of Gene Regulation, Institute for Advanced Medical Research, Keio University School of Medicine, Tokyo, Japan

**Keywords:** SARS-CoV-2, COVID-19, rapid antibody test, IgG, IgM

## Abstract

**Background:**

The usability of laboratory tests related to severe acute respiratory syndrome coronavirus 2 (SARS-CoV-2) is critically important for the world undergoing the COVID-19 pandemic. The present study aimed to assess the diagnostic usability of rapid tests for the detection of antibody against SARS-CoV-2 through comparison of their results with the results of reverse transcription polymerase chain reaction (RT-PCR) test for the detection of SARS-CoV-2 genomic RNA and with the results of a quantitative test for antibody detection.

**Methods:**

Serum samples were collected from 18 patients undergoing RT-PCR testing for SARS-CoV-2. Twelve patients were RT-PCR positive while six were negative. A quantitative test based on chemiluminescent immunoassay and three rapid tests based on immunochromatography were performed to detect anti-SARS-CoV-2 IgG and IgM.

**Results:**

All the antibody tests exhibited poor sensitivity at the timing of initial RT-PCR diagnosis. IgG responses occurring prior to or simultaneously with IgM responses were observed through not only the quantitative test but also the three rapid tests. Based on concordance with the quantitative test results, the large variance among the three rapid tests was revealed.

**Conclusions:**

All antibody tests were unsatisfactory to replace RT-PCR for the early diagnosis of COVID-19. Rapid antibody tests as well as a quantitative antibody test were useful in the assessment of immune responses in COVID-19. The obvious variance among the three rapid tests suggested limited accuracy and difficult standardization. Diagnostic usability of rapid antibody tests for COVID-19 should be investigated rigorously.

## Introduction

COVID-19, an infectious disease due to severe acute respiratory syndrome coronavirus 2 (SARS-CoV-2) is mainly diagnosed through viral RNA detection by reverse transcription polymerase chain reaction (RT-PCR) testing of nasal or pharyngeal swabs, saliva, or sputum. RT-PCR tests require equipment, reagents and well-trained medical technologists. The availability of such resources is limited. In addition, sampling of nasal or pharyngeal swabs may run the risk of exposing medical staff to viruses.

In contrast, rapid antibody tests to detect blood, plasma or serum IgG and IgM specific to SARS-CoV-2 require neither special equipment nor training. Turnaround time is far shorter than RT-PCR tests. Several reports demonstrated high diagnostic accuracy of rapid antibody tests by comparing them with RT-PCR tests using samples from hospitalized patients who were already diagnosed as COVID-19 according to RT-PCR test results.^1,2^ However, the usability of rapid tests for antibody detection as early diagnostic methods remains to be ascertained. In order to ascertain whether rapid antibody tests substitute for RT-PCR tests, we assessed the diagnostic usability of several commercially available rapid antibody tests using the serum samples obtained at the timing of RT-PCR diagnosis. We also evaluated their accuracy according to concordance with a quantitative test for IgG and IgM specific to SARS-CoV-2 as the standard reference.

## Materials and methods

### Samples

The present study was performed under the approval by the Ethics Committee of Keio University School of Medicine (20190339, 20200036, and 20200059).

Serum samples for the present study were collected from 18 patients undergoing RT-PCR testing for SARS-CoV-2 at Keio University Hospital (Tokyo, Japan). Twelve patients were RT-PCR positive while six were negative. One-Step Real-Time RT-PCR assays were performed using BD MAX with BD MAX TNA MMK and BD MAX ExK TNA-3 (Becton Dickenson, Franklin Lakes, NJ, USA). The primers and the probes specific to SARS-CoV-2 N1 and N2 were used for RT-PCR. The RT-PCR positivity was defined as threshold cycle (Ct) values being less than 45 cycles.

Serum samples, which were collected from the patients on the day of RT-PCR using nasopharyngeal specimens or on the day before or after the testing, were used for their laboratory tests. After the performance of tests, the residual serum of each patient was subjected to evaluation of the usability of antibody tests as early diagnostic methods, for which RT-PCR results were used as the standard reference regarding COVID-19 diagnosis. In addition, the residuals of serum samples serially collected from RT-PCR positive patients at various time points within three weeks after the RT-PCR performance were subjected to evaluation of the accuracy of rapid antibody tests according to concordance with the quantitative antibody test.

### Rapid antibody tests and a quantitative antibody test

The rapid tests for the detection of IgG and IgM specific to SARS-CoV-2 were done using ALLTest 2019-nCoV IgG/IgM Rapid Test Cassette (ALLTest) (Hangzhou AllTest Biotech, Hangzhou, China), STANDARD Q COVID-19 IgM/IgG Duo Test (SD Biosensor) (SD BIOSENSOR, Suwon, Republic of Korea) and Kurabo Rapid immunochromatographic test for detecting the antibody of SARS-CoV-2 (KURABO) (Kurabo, Osaka, Japan). These three kits are based on immunochromatography.

A qualitative antibody test based on chemiluminescence immunoassay was done using the iFLASH Immunoassay Analyzer and the SARS-CoV-2 IgG and IgM reagents (iFLASH) (Shenzhen YHLO Biotech, Shenzhen, China). With respect to IgG and IgM, the titre of 10 AU/mL was the cut-off value to distinguish positivity and negativity.

### Analysis

Age, sex and results of rapid antibody tests and a quantitative antibody test were subjected to comparison between RT-PCR positive and negative patients (Table 1). Fisher’s exact test for categorical values and Mann-Whitney U test for serial values were done.

**Table 1.** Background of patients with antibody test results at the timing of initial RT-PCR diagnosis.

	PCR positive	PCR negative	
	(*n* = 12)	(*n* = 6)	
Age, IQR	61 (51.5–72.5)	56 (41–78)	*P* = 1.00
Gender (male/female)	9/3	3/3	*P* = 0.34
Severity of COVID-19^a^			
Asymptomatic	2 (16.7%)	–	
Mild	7 (58.3%)	–	
Moderate	2 (16.7%)	–	
Severe	1 (8.3%)	–	
Days after onset^b^	4 (0–10)	–	
Ct values of PCR			
N1 gene	22.4 (17.0–42.9)	–	
N2 gene	14.6 (9.0–30.0)	–	
Immunosuppression			
Steroid use	0 (0.0%)	–	
Immunosuppressant use	1 (8.3%)	–	
Diabetes mellitus	0 (0.0%)	–	
Cancer chemotherapy	1 (8.3%)	–	
ALLTest IgM	2/10^b^	0/6^b^	*P* = 0.53
IgG	1/11^b^	0/6^b^	*P* = 1.00
SD BIOSENSOR IgM	1/11^b^	0/6^b^	*P* = 0.53
IgG	2/10^b^	0/6^b^	*P* = 1.00
KURABO IgM	0/12^b^	0/6^b^	–
IgG	2/10^b^	0/6^b^	*P* = 0.53
Quantitative antibody test (iFLASH)			
IgM	1/11^b^	0/6^b^	
IgM titre (AU/mL, IQR)	0.71 (0.36–0.86)	0.24 (0.19–0.47)	*P* = 0.62
IgG	3/9^b^	0/6^b^	
IgG titre (AU/mL, IQR)	1.10 (0.76–6.33)	0.40 (0.37–0.69)	*P* = 0.13

^a^The severity at the timing of diagnostic PCR performance is shown. Definition of severity is as follows: ‘mild’ for symptomatic patients without hypoxia, ‘moderate’ for symptomatic patients with hypoxia requiring oxygen inhalation and ‘severe’ for symptomatic patients with hypoxia requiring mechanical ventilation.

^b^For symptomatic patients, the onset date was defined as the date on which symptom of COVID-19 or radiographic abnormality related to COVID-19 appeared. For asymptomatic patients without significant radiographic abnormality, the onset date was defined as the date on which the initial PCR positivity was confirmed. Antibody test results at the timing of initial RT-PCR diagnosis are displayed as follows: number of positive test results/number of negative test results.

For RT-PCR positive patients, severity of COVID-19 (mild: symptomatic patients without hypoxia; moderate: symptomatic patients with hypoxia requiring oxygen therapy and severe: symptomatic patients with hypoxia requiring mechanical ventilation), Ct values of RT-PCR for SARS-CoV-2 N1 and N2 and days from the onset to the sampling were summarized. The onset date was basically defined as the date on which symptoms (fever, cough, sputum, dyspnoea, olfactory taste disorder and/or sore throat) or chest imaging findings compatible with COVID-19 such as bilateral ground glass opacity in chest computed tomography (CT) appeared. In the case of asymptomatic patients, the onset date was instead defined as the date on which RT-PCR positivity was confirmed.

The numbers of positivity and negativity regarding antibody test results using the residuals of serum samples serially collected from PCR positive patients were summarized for each day after the onset for assessing seroconversion. In order to compare the accuracy among rapid antibody test kits, concordance rates, sensitivity, specificity, positive predictive values and negative predictive values were evaluated using quantitative antibody test results. Kappa coefficients were calculated to measure concordance. The threshold antibody titres defined as the minimum quantitative antibody titres in which both the quantitative antibody test and each rapid antibody test exhibit positivity were detected. The rates of occurrence of positive results of rapid antibody tests over the threshold antibody titres were calculated. For statistical analysis, SPSS 25 (IBM, Armonk, NY, USA) was used, and a *P* value < 0.05 was considered significant.

## Results

### Accuracy of antibody tests at the timing of RT-PCR diagnosis

Of 12 RT-PCR positive patients, 7 had mild symptoms, 2 had moderate symptoms, and 1 had severe symptoms at the timing of diagnostic PCR performance (Table 1). Two asymptomatic patients underwent RT-PCR testing because of being high-risk contacts. The median of days after the onset was four days. The Ct values of RT-PCR were 30 cycles or less in the subjects except an asymptomatic patient. Of six RT-PCR negative patients, three contracted community acquired pneumonia (CAP) and three had a transient fever without infection foci suspected clinically. All CAP patients got afebrile and had their symptoms resolved with antibiotics. The transient fever in three patients disappeared at the next day of RT-PCR testing. While the specificity for all three rapid antibody test kits was 100%, the sensitivity was 20% or lower. The quantitative IgG test positivity was observed in three patients.

**Figure 1.**
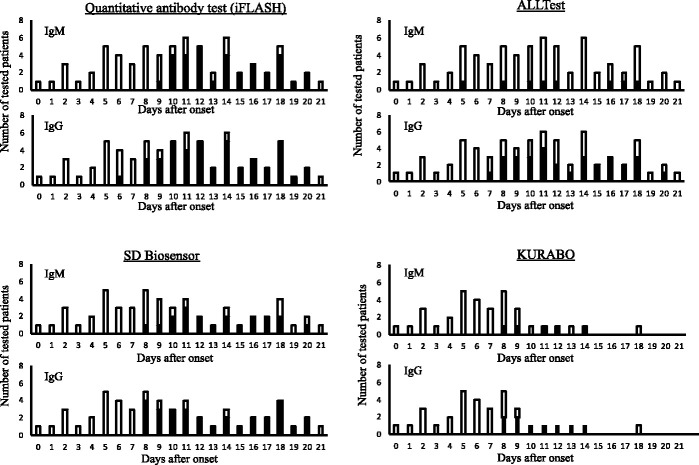
The positivity of each antibody test kit at various time points after the onset reveals distinct seroconversion. The number of tested patients and days after the onset are shown. Black columns indicate patients with positive results while white columns indicate patients with negative results. For 10 symptomatic patients, the onset date was defined as the date on which symptoms or chest imaging findings compatible with COVID-19 appeared. For two asymptomatic patients lacking significant chest imaging findings, the onset date was defined as the date on which RT-PCR positivity was confirmed.

**Figure 2.**
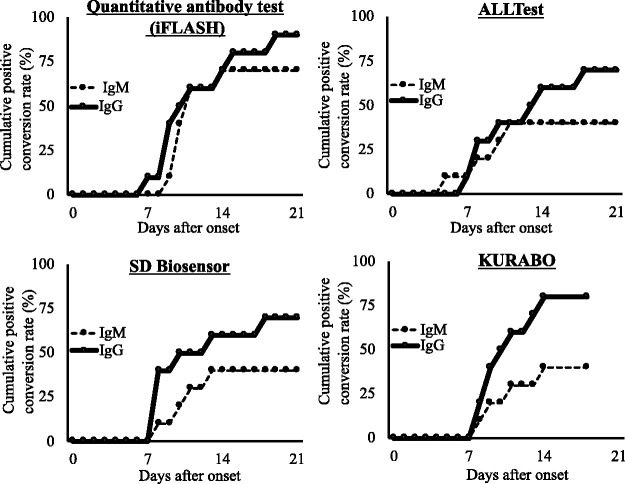
Relation between the cumulative positive conversion rates of ten symptomatic patients and days after symptom onset reveals that IgM seroconversion did not precede IgG seroconversion. Calculation of the cumulative positive conversion rates was based on the numbers of patients, who exhibited the positivity at least one time, at each time point after the onset.

All samples exhibiting the positivity were collected after six days or more since the onset (Figure 1, Table S1 and Table S2). While most of the IgM and IgG test results from samples collected within the first week since the onset were negative, the IgG positivity prevailed in eight or nine days after the onset in not only the quantitative test but also all three rapid tests, which were performed for samples collected serially over 10 days after the onset. On the other hand, the IgM positivity prevailed in 10 days after the onset in the quantitative test and the rapid test using SD BIOSENOSOR kit, following the prior appearance of IgG positivity. Of note, such observations were not obtained by ALLTest kit. Although the IgM positivity seemed to prevail in 11 days after the onset also in the rapid test using KURABO kit, the samples were fewer than those tested using other kits to make it difficult to put an interpretation on the observations. Taken together, according to our observations, IgM seroconversion did not precede IgG seroconversion as proved by the cumulative positivity rates regarding IgG and IgM (Figure 2).

### Accuracy of rapid antibody tests according to concordance with the quantitative test

Regarding detection of anti-SARS-CoV-2 IgG, statistical significance was observed in all three rapid test kits using the quantitative test results as the standard reference (Table 2). SD BIOSENSOR kit exhibited the highest concordance with the quantitative test (*k *=* *0.93). Regarding detection of anti-SARS-CoV-2 IgM, statistical significance was observed in SD BIOSENSOR and KURABO kits but not in ALLTest kit. SD BIOSENSOR kit exhibited the highest concordance with the quantitative test (*k *=* *0.76). Overall, the accuracy of anti-SARS-CoV-2 IgM tests was apparently lower than that of anti-SARS-CoV-2 IgG.

**Table 2.** Accuracy of results from rapid antibody tests regarding concordance with quantitative antibody test results.

	ALLTest		SD BIOSENSOR		KURABO	
	(*n* = 69)		(*n* = 55)		(*n* = 34)	
IgG antibody
Concordance rate (%)	76.8	*k* = 0.55	96.4	*k* = 0.93	88.2	*k* = 0.72
Sensitivity (%)	67.4	*χ*^2^ = 23.38	96.7	*χ*^2^ = 47.23	72.7	*χ*^2^ = 17.88
Specificity (%)	92.3	*P* < 0.01	96	*P* < 0.01	95.7	*P* < 0.01
Positive predictive value (%)	93.5		96.7		88.9	
Negative predictive value (%)	64.2		96		88	
Threshold IgG titre (AU/mL)	19.1		19.1		19.1	
Test positive rate over IgG threshold titre	69		100		100	
IgM antibody
Concordance rate (%)	55.1	*k* = 0.07	88.7	*k* = 0.76	91.2	*k* = 0.62
Sensitivity (%)	15.2	*χ*^2^ = 0.78	85	*χ*^2^ = 31.34	75	*χ*^2^ = 13.14
Specificity (%)	91.7	*P* = 0.47	90.9	*P* < 0.01	93.3	*P* < 0.01
Positive predictive value (%)	62.5		85		60	
Negative predictive value (%)	54.1		90.9		96.6	
Threshold IgM titre (AU/mL)	13.8		12.2		12.2	
Test positive rate over threshold titre	17.9		89.5		75	

## Discussion

PCR-based methods for SARS-CoV-2 detection are not only inapplicable to point of care testing because of requiring specialized instruments, labour and time but also are known to have variation in false-negative rate by time since exposure.^3^ Although rapid tests are expected to overcome such concerns, their accuracy seemingly varies by manufacturers. The present study aimed to assess the diagnostic performance and accuracy of immunochromatography-based rapid test kits for antibody detection through comparison with RT-PCR for SARS-CoV-2 as well as with chemiluminescent immunoassay-based quantitative analysis of antibodies.

Zhao et al. reported antibody detection one day after the symptom onset by ELISA kit targeting at S1 protein.^4^ In contrast, both the rapid tests and the quantitative test exhibited poor sensitivity at the timing of initial RT-PCR diagnosis in the present study. Antibodies became detectable in about eight days after the onset. These observations are consistent with previous reports demonstrating that antibody test positivity proved COVID-19 occurrence in the middle or late stage of the disease.^2,5,6^ In addition, it has been reported that use of antibody tests without RT-PCR tests in a first week of symptomatic phase, in which the higher rate of viral shedding is observed than in the later phase, results in failure to make diagnosis of COVID-19.^7^

The unique antibody responses to SARS-CoV-2, such as IgM seroconversion not preceding IgG seroconversion, have been reported in previous studies.^8,9^ Consistent with these reports, IgG responses occurring prior to or simultaneously with IgM responses were observed through not only the quantitative test but also the rapid tests in the present study. There are at least two possibilities to explain such responses. One is reinfection or primary infection with SARS-CoV-2 following prior infection with other coronaviruses to lead to cross-reactive immune. Another possibility is non-canonical immune responses to primary viral infection, which may reportedly occur independently of germinal centre formation in peripheral lymphoid organs.^10,11^ In this case, a time length resulting from the occurrence of canonical class-switch from IgM to IgG in germinal centres is absent. IgG responses might be allowed to appear prior to or simultaneously with IgM responses.

Comparison with the quantitative test revealed the variance of the rapid antibody tests to allow us to recognize their limited accuracy and difficult standardization. Despite the fact that all the three rapid tests target SARS-CoV-2 nucleocapsid protein, the SD Biosensor IgG test exhibited great concordance with the quantitative test while the ALLTest IgM test exhibited poor concordance. The poor sensitivity of ALLTest kits has also been reported in a previous study.^12^ The accuracy of IgM tests totally seemed to be lower than that of IgG tests. Rheumatoid factor existing in serum IgM is known to interfere with immunological assays via binding to the IgG Fc portion.^13^ IgM also contains natural antibodies reactive to various substances, which may bind to the components of the test kits to interfere with the assay.^14^ These mechanisms might be related to the difference in the accuracy between IgG and IgM tests.

While a number of studies evaluating rapid antibody tests for the detection of SARS-CoV-2 infection have been published, the advantage of the present study is to compare results of the various rapid antibody tests not only with results of the PCR test but also with results of the quantitative antibody test to reveal the limited accuracy and difficult standardization. This would lead to additional impact on the understanding of the diagnostic potential of rapid antibody tests.^15,16^ The large variance regarding the concordance with the quantitative antibody test leads us to consider about how best to deal with data that are collected using various rapid tests. While the present study focused on diagnostic application of rapid antibody tests for COVID-19, epidemiological application to assessment of herd immunity against SARS-CoV-2 infection is also of critical importance.

Although the true negative results and the cumulative positive conversion rate are shown in Table 1 and Figure 2, respectively, the number of tests in the present study was insufficient to refer to them as the specificity and the sensitivity for iFLASH. According to a recent study, the specificity and the sensitivity for iFLASH-IgG were 100% and 92.9%, respectively by using the cut-off provided by the manufacturer, which was also used in the present study. The specificity and the sensitivity for iFLASH-IgM were 98.7% and 62.2%, respectively.^17^

While the SD Biosensor seemed to be better at detecting anti-SARS-CoV-2 IgM than the ALLTest according to the results, it remains to be assessed whether the SD Biosensor had higher false positivity in the present study. In a recent study, the specificity for the SD Biosensor-IgG and the specificity for the Alltest-IgG were both 99.0% while that for the SD Biosensor-IgM and that for the Alltest-IgM were both 98.0%.^18^ This suggests that there are no differences in false positivity between the SD Biosensor and the ALLTest.

As described above, natural antibodies contained in the IgM fraction might react to some impurities potentially existing in kits to interfere with immunoassays.^14^ Rheumatoid factors may also give rise to overestimation or underestimation of immunoassays.^13^ It is plausible that such interference is expected to result in the difference in the results among various rapid antibody tests. However, it is difficult to address what is the cause of the difference because the details of the components of each kit have not yet been disclosed.

As observed in the present study, the recent studies have suggested that IgM tests for detecting SARS-CoV-2 infection exhibit low reliability because of large variance among various kits as well as insufficient specificity or sensitivity. On the other hand, IgG tests seem to be more reliable because of small variance as well as higher specificity and sensitivity.^18–20^

While the merit of the present study lies in effective estimation of multiple serodiagnostics using a minimum number of clinical subjects, it also, in turn, reflect the limitations of the present study including imprecise estimates due to heterogeneity in the timing for sampling at which results were obtained, as well as a small number of patients and samples. To address the concerns, basic, laboratory and epidemiological findings should be accumulated relevantly to diagnostics and therapeutics.

In conclusion, all antibody tests were unsatisfactory to replace RT-PCR for SARS-CoV-2 as early diagnostic methods while being useful in the assessment of immune responses in COVID-19. Diagnostic usability of rapid antibody tests should be investigated rigorously in consideration of their variance.

## Supplemental Material

sj-pdf-1-acb-10.1177_0004563220984827 - Supplemental material for Evaluation of the usability of various rapid antibody tests in the diagnostic application for COVID-19 Click here for additional data file. Supplemental material, sj-pdf-1-acb-10.1177_0004563220984827 for Evaluation of the usability of various rapid antibody tests in the diagnostic application for COVID-19 by Yoshifumi Uwamino, Masatoshi Wakui, Wataru Aoki, Toshinobu Kurafuji, Emmy Yanagita, Maasa Morita, Mika Nagata, Rika Inose, Masayo Noguchi, Hiromitsu Yokota, Naoki Hasegawa, Hideyuki Saya, Mitsuru Murata and for the Keio Donner Project Team in Annals of Clinical Biochemistry

sj-pdf-2-acb-10.1177_0004563220984827 - Supplemental material for Evaluation of the usability of various rapid antibody tests in the diagnostic application for COVID-19 Click here for additional data file. Supplemental material, sj-pdf-2-acb-10.1177_0004563220984827 for Evaluation of the usability of various rapid antibody tests in the diagnostic application for COVID-19 by Yoshifumi Uwamino, Masatoshi Wakui, Wataru Aoki, Toshinobu Kurafuji, Emmy Yanagita, Maasa Morita, Mika Nagata, Rika Inose, Masayo Noguchi, Hiromitsu Yokota, Naoki Hasegawa, Hideyuki Saya, Mitsuru Murata and for the Keio Donner Project Team in Annals of Clinical Biochemistry
