# Supplementary material for: Evaluation of the usability of various rapid antibody tests in the diagnostic application for COVID-19
Source: Ann Clin Biochem. 2021 Jan 7;58(3):174–80. doi: 10.1177/0004563220984827 (PMC7797350; doi:10.1177/0004563220984827)
Supplement: sj-pdf-1-acb-10.1177_0004563220984827 - Supplemental material for Evaluation of the usability of various rapid antibody tests in the diagnostic application for COVID-19 [file sj-pdf-1-acb-10.1177_0004563220984827.pdf]

Table S1: Antibody test results of 12 patients

Quantitative antibody test (iFLASH)

IgM

Days after onset	0	1	2	3	4	5	6	7	8	9	10	11	12	13	14	15	16	17	18	19	20	21
1		○				○													○			
2			○			○				○		○			○							○
3			○																			
4											●	●	●		●	●	●	●	●	●	●	
5								○	○					○	●				●		●	
6	○				○	○							●									
7					○		○	○	○	●	●	●										
8			○						○	○	○	○					●	●	●			
9				○		○	○	○	○	○	●	●	●	●	●	●	●					
10							○					●	●						●			
11						○	○								○							
12									○		●		●		●							

IgG

Days after onset	0	1	2	3	4	5	6	7	8	9	10	11	12	13	14	15	16	17	18	19	20	21
1		○				○													●			
2			○			○				○		○			○							○
3			○																			
4											●	●	●		●	●	●	●	●	●	●	
5								○	○					●	●				●		●	
6	○				○	○							●									
7					○		○	○	●	●	●	●										
8			○						○	●	●	●					●	●	●			
9				○		○	○	○	●	●	●	●	●	●	●	●	●					
10							●					○	●						●			
11						○	○								●							
12									●		●		●		●							

●-positive test, ○-negative test

ALL test

IgM

Days after onset	0	1	2	3	4	5	6	7	8	9	10	11	12	13	14	15	16	17	18	19	20	21
1		○				○													○			
2			○			○				○		○			○							○
3			○																			
4											●	○	○		○	○	○	○	○	○	○	
5								○	○					○	○				○		○	
6	○				○	○							●									
7					○		○	○	●	○	○	○										
8			○						○	○	○	●					●	●	●			
9				○		○	○	○	○	○	○	○	○	○	○	○	○					
10							○					○	○						○			
11						●	○								○							
12									○		○		○		○							

IgG

Days after onset	0	1	2	3	4	5	6	7	8	9	10	11	12	13	14	15	16	17	18	19	20	21
1		○				○													●			
2			○			○				○		○			○							○
3			○																			
4											●	●	●		●	●	●	●	○	○	○	
5								○	○					●	●				●		●	
6	○				○	○							●									
7					○		○	○	●	●	●	●										
8			○						●	●	●	●					●	●	●			
9				○		○	○	●	●	●	○	●	○	○	○	●	●					
10							○					○	○						○			
11						○	○								●							
12									○		○		○		○							

●-positive test, ○-negative test

SD Biosensor

IgM

Days after onset	0	1	2	3	4	5	6	7	8	9	10	11	12	13	14	15	16	17	18	19	20	21
1		○				○													○			
2			○			○				○		○			○							○
3			○																			
4											●	●	●		●	●	●	●	○	○	○	
5								○	○					●	●				●		●	
6	○				○	○							●									
7					○		○	○	●	●	●	●										
8			○						○	○	○	●					●	●	●			
9				○		○	○	○	○	○												
10							○															
11						○																
12									○													

IgG

Days after onset	0	1	2	3	4	5	6	7	8	9	10	11	12	13	14	15	16	17	18	19	20	21
1		○				○													●			
2			○			○				○		○			○							○
3			○																			
4											●	●	●		●	●	●	●	●	●	●	
5								○	○					●	●				●		●	
6	○				○	○							●									
7					○		○	○	●	●	●	●										
8			○						●	●	●	●					●	●	●			
9				○		○	○	○	●	●												
10							○															
11						○	○															
12									●													

●-positive test, ○-negative test

KURABO

IgM

Days after onset	0	1	2	3	4	5	6	7	8	9	10	11	12	13	14	15	16	17	18	19	20	21
1		○				○													○			
2			○			○				○												
3			○																			
4											○											
5								○	○					○								
6	○				○	○							●									
7					○		○	○	○	●												
8			○						○	○												
9				○		○	○	○	●													
10							○					●										
11						○	○								●							
12									○													

IgG

Days after onset	0	1	2	3	4	5	6	7	8	9	10	11	12	13	14	15	16	17	18	19	20	21
1		○				○													○			
2			○			○				○												
3			○																			
4											●											
5								○	○					●								
6	○				○	○							●									
7					○		○	○	○	●												
8			○						○	●												
9				○		○	○	○	●													
10							○					●										
11						○	○								●							
12									●													

●-positive test, ○-negative test
