# Supplementary material for: Evaluation of the usability of various rapid antibody tests in the diagnostic application for COVID-19
Source: Ann Clin Biochem. 2021 Jan 7;58(3):174–80. doi: 10.1177/0004563220984827 (PMC7797350; doi:10.1177/0004563220984827)
Supplement: sj-pdf-2-acb-10.1177_0004563220984827 - Supplemental material for Evaluation of the usability of various rapid antibody tests in the diagnostic application for COVID-19 [file sj-pdf-2-acb-10.1177_0004563220984827.pdf]

Table S2: Antibody titers measured by iFLASH and COVID-19 severity during the clinical course.

iFLASH IgM

	Age	Gender	Severity*	0	1	2	3	4	5	6	7	8	9	10	11	12	13	14	15	16	17	18	19	20	21
1	20s	F	mild		0.86				0.81													1.27			
2	60s	M	mild			0.86			0.81				0.82		0.87			0.78							0.97
3	20s	F	mild			0.78																			
4	60s	M	severe											13.75	31.16	31.87		23.6	18.38	16.63	14.41	13.22	12.21	11.09	
5	60s	F	moderate								0.18	0.19					2.36	12.47				24.6		32.16	
6	80s	M	severe	0.64				1.12	1.75							73.76									
7	70s	M	severe					0.26		0.37	0.81	2.73	12.22	22.49	28.12										
8	70s	M	severe			0.32						0.43	0.63	0.96	2.13					40.45	42.13	39.92			
9	60s	M	severe				0.44		0.37	0.37	0.45	1.2	8.65	13.83	14.46	22.22	20.05	16.24	35.51	34.09					
10	60s	M	severe							0.39					14.22	23.37						24.98			
11	80s	M	severe						0.84	0.81								9.33							
12	40s	M	moderate									9.18		15.9		19.46		18.64							

iFLASH IgG

	Age	Gender	Severity*	0	1	2	3	4	5	6	7	8	9	10	11	12	13	14	15	16	17	18	19	20	21
1	20s	F	mild		0.64				0.65													28.35			
2	60s	M	mild			0.41			0.4				0.39		0.37			0.35							0.41
3	20s	F	mild			4.88																			
4	60s	M	severe											57.17	93.53	97.64		117.32	83.98	98.37	89.34	94.12	80.29	63.53	
5	60s	F	moderate								0.78	0.75					23.34	86.06				90.56		69.62	
6	80s	M	severe	0.86				0.74	0.86							57.84									
7	70s	M	severe					0.68		0.94	4.58	23.42	62.79	75.52	57.07										
8	70s	M	severe			0.89						7.97	19.07	35.78	51.97					70.73	76.29	68.76			
9	60s	M	severe				1.31		0.89	1.51	3.18	21.03	48.19	51.3	42.89	42.55	44.57	45.67	42.81	46.94					
10	60s	M	severe							10.66					7.15	51.6						62.07			
11	80s	M	severe						2.89	2.41								67.95							
12	40s	M	moderate									44.74		49.49		51.61		61.91							

*The maximum severity during clinical course is shown. Definition of seveity is as follows: “mild” for symptomatic patients without hypoxia, “moderate” for symptomatic patients with hypoxia requiring oxygen inhalation, and “severe” for symptomatic patients with hypoxia requiring mechanical ventilation
